# Differential Neural Responses to Food Images in Women with Bulimia versus Anorexia Nervosa

**DOI:** 10.1371/journal.pone.0022259

**Published:** 2011-07-20

**Authors:** Samantha J. Brooks, Owen G. O′Daly, Rudolf Uher, Hans-Christoph Friederich, Vincent Giampietro, Michael Brammer, Steven C. R. Williams, Helgi B. Schiöth, Janet Treasure, Iain C. Campbell

**Affiliations:** 1 Department of Neuroscience, Uppsala University, Uppsala, Sweden; 2 Department of Neuroimaging, Centre for Neuroimaging Sciences, King's College London Institute of Psychiatry, London, United Kingdom; 3 Section of Eating Disorders, Department of Psychological Medicine, King's College London Institute of Psychiatry, London, United Kingdom; 4 Psychosomatic and General Internal Medicine, Centre for Psychosocial Medicine, Heidelberg, Germany; Royal Holloway, University of London, United Kingdom

## Abstract

**Background:**

Previous fMRI studies show that women with eating disorders (ED) have differential neural activation to viewing food images. However, despite clinical differences in their responses to food, differential neural activation to thinking about eating food, between women with anorexia nervosa (AN) and bulimia nervosa (BN) is not known.

**Methods:**

We compare 50 women (8 with BN, 18 with AN and 24 age-matched healthy controls [HC]) while they view food images during functional Magnetic Resonance Imaging (fMRI).

**Results:**

In response to food (vs non-food) images, women with BN showed greater neural activation in the visual cortex, right dorsolateral prefrontal cortex, right insular cortex and precentral gyrus, women with AN showed greater activation in the right dorsolateral prefrontal cortex, cerebellum and right precuneus. HC women activated the cerebellum, right insular cortex, right medial temporal lobe and left caudate. Direct comparisons revealed that compared to HC, the BN group showed relative deactivation in the bilateral superior temporal gyrus/insula, and visual cortex, and compared to AN had relative deactivation in the parietal lobe and dorsal posterior cingulate cortex, but greater activation in the caudate, superior temporal gyrus, right insula and supplementary motor area.

**Conclusions:**

Women with AN and BN activate top-down cognitive control in response to food images, yet women with BN have increased activation in reward and somatosensory regions, which might impinge on cognitive control over food consumption and binge eating.

## Introduction

Bulimia Nervosa (BN) is defined by recurrent episodes of binge eating of large amounts of food, and compensatory measures to control for weight gain. (Diagnostic and Statistical Manual Fourth Edition, [1]. People with BN have deficient self-regulatory control, represented at the neural level by reduced prefrontal, and increased mesolimbic responses [2], [3]. Furthermore, neural activation to food stimuli in women with BN is consistently observed in motor regions [2], [4], [5], however, the nature of activation in reward regions is inconclusive. One hypothesis is that hypoactivation in mesolimbic reward regions underlies binge eating [6]. However, an alternative hypothesis is that there is hyperactivation of reward and somatosensory neural systems in people who binge [7], [8]. One way to test these hypotheses is to compare neural activation to food images in people who have eating behaviours on the extremes of cognitive inhibition of appetite (AN) versus reduced inhibition of appetite (BN).

Those with BN report an excessive urge to eat, coinciding with a sense of lack of control over their eating. This is in contrast to anorexia nervosa (AN), which is defined by emaciation and maintainance of less than eighty-five percent normal body weight via deliberate control of food intake and inhibition of appetite. However, women with both AN and BN report a fear of gaining weight and a desire to be thinner. These differences may manifest at the neural level as differential activation when thinking about eating food. People who binge-eat show altered prefrontal-cortical-striatal-insula responses to appetitive stimuli [7], [9]. One fMRI study compared neural activation in women with AN and BN who passively viewed images of food, finding reduced prefrontal cortex but greater cerebellum activation in women with BN [3]. However, no fMRI study to date has attempted to deliberately evoke cognitions associatied with eating in women with AN and BN, and to measure neural responses to food images.

The aim of this exploratory study, which uses a novel paradigm whereby eating-related cognitions are evoked by thinking about eating food shown in images, is to demonstrate a differential pattern of activation in cortico-striatal-insula regions between women currently ill with AN and BN. We hypothesize that: a) women with AN and BN compared to healthy controls (HC) have reduced striatal-insular cortex, and greater prefrontal cortical activation when thinking about eating food versus non-food items shown in images; b) women with BN have reduced prefrontal cortical activation but greater striatal-insular cortex responses in comparison to women with AN.

## Methods

### Ethics statement

This study was approved by the South London and Maudsley (SLaM) NHS Trust Ethics Committee, study number: 297/02. Additionally, the study adhered to the guidelines as set out in the Declaration of Helsinki. Written informed consent was required from all participants, as approved by the SLaM ethics committee, and they were reimbursed for their participation.

### Participants

50 right-handed females (aged 16–50 years) volunteered to participate in the study: 8 had a current DSM-IV diagnosis of bulimia nervosa (BN), 18 had a current diagnosis of anorexia nervosa (AN). Of these, 11 had a diagnosis of *restricting* AN (RAN), whereas 7 had a diagnosis of *binge purging* AN (BPAN). 24 were healthy control (HC) age-matched females with no history of psychiatric disorder and who were in the normal weight range. HC were recruited from college students who responded to an advertisement (more details of the AN and HC groups are found in unpublished data, Brooks et al., in preparation). Women recruited with BN were receiving treatment from the Outpatients Unit of the South London and Maudsley (SLaM) NHS Trust, and their duration of illness was on average 12 years (s.d. 6.3). Those with AN were being treated in the Inpatient Unit: the total duration of their illness was on average 7 years (s.d. 4.0). The total duration of illness for the RAN subgroup was 9 years (s.d. 7.4), and also 9 years for the BPAN subgroup (s.d. 5.0). Diagnosis of BN or AN was made using the Structured Clinical Interview for DSM-IV (SCID; [10]) and confirmed by a psychiatrist. Self report questionnaires were completed by all participants before the experiment. The subjects were matched for age and IQ, and were asked to confirm that they had eaten a normal lunch before the scan, and that they had not eaten anything or drank anything containing caffeine for at least two hours, or alcohol for 24 hours prior to the scan. For all participants, exclusion criteria were a history of head trauma, hearing or visual impairments, neurological disease, metallic implants, claustrophobia or psychotropic medication other than selective serotonin reuptake inhibitors (SSRIs). Due to technical difficulties 2 women with BN, 5 with AN and 3 HC were excluded: thus, 8 women with BN, 18 with AN (11 with RAN, 7 with BPAN) and 24 HC contributed to the analysis.

### Stimuli

72 colour photographs of high and low calorie, sweet and savoury food were presented on white plates on a blue background in random order: these were created by the authors. The control condition was 72 colour photographs of non-food items on white plates on a blue background (created by the authors, an example is found in the online supplement). Food and non-food items were selected and matched according to colour and visual structure. All images are available on request.

### Questionnaire Measures

#### The Eating Disorders Examination – Questionnaire, EDEQ [11]

This is a 36-item measure of dysfunctional behaviour and cognitions related to eating, with sub-scales: eating concern, shape concern, weight concern and restrained eating, together with a global eating disorder score. Questions are scored between 0–6: higher scores indicate greater eating disorder pathology.

#### The Hospital Anxiety and Depression Scale, HADS [12]

This is a 14-item self-report measure with 7 related to anxiety and 7 related to depression. Individual questions are scored on a 4-point scale: higher scores indicate greater anxiety or depression.

#### Structured Clinical Interview for Diagnosis-Researcher Version SCID-R [13]

This structured interview is used for diagnosis, for general screening, and to obtain demographic information. Duration of illness is the time between diagnosis of AN and the time of the scan. It is noted that symptoms are most likely present before the formal diagnosis, but this measure gives a systematic score of illness duration.

### Procedure

Scanning was performed between 1.30 and 4 pm. Images were presented on a rear-projection screen and viewed through a double-mirror periscope attached to the headcoil. Images (food versus non-food) were presented during the same scanning period. An AB block-design of 6 blocks for the active (food images [A]) and 6 blocks for the control (non-food images [B]) conditions was used: blocks were alternated between active and control conditions. Each block consisted of 12 images: each was presented for 3 sec. with no gap, ie images for each category were presented continuously for 36 seconds. At the beginning of each block, a ‘partially silent’ period of 8 seconds, and another 8 sec. partial silent period at the end of each block (where no data was acquired and the Echo Planar Image [EPI] readout was disabled) was used to present audio stimuli/ obtain verbal responses. During these periods, slice selection, Radio Frequency (RF) and gradients continued, in order to maintain the MR signal in a steady state and to allow data collection to continue during subsequent volumes. Pre-recorded audio stimuli asked participants to a) imagine eating the food in the images, and b) imagine using the non food items. For each instruction, 4 separate but semantically similar phrases were given via headphones. In the second partially-silent period (at the end of each block), participants were asked to rate how anxious they felt on a scale of 0–10: participants reponded verbally. The duration of each block was 52 seconds (36 seconds of stimuli + two 8 second periods of ‘partial silence’), repeated 12 times (food versus non-food): total duration of the presentation of food versus non-food images was therefore 10.4 minutes.

### Image acquisition

The fMRI acquisition was performed on a GE Signa 1.5-Tesla scanner (GE Medical Systems, Milwaukee, Wisconsin). T2* -weighted images depicting Blood Oxygen Level Dependent (BOLD) contrast were acquired with a TR of 4 sec. (repetition time) with an in-plane resolution of 3.75 mm×3.75 mm. The echo time was 40 msec and the flip angle was 90°. Whole brain coverage was acquired in 43 slices (slice thickness 3 mm, interslice gap 0.3 mm). Fifty-four T2* -weighted whole brain volumes were acquired in each of the two conditions in both experiments.

### Statistical analysis

Data was analyzed with the XBAM software developed at the Institute of Psychiatry [14], [15], [16]. Non-parametric analysis was used as group fMRI data is often not normally distributed [17]. Following motion correction, the estimated BOLD effect was modelled by two Poisson functions with haemodynamic delays of 4 and 8 sec. The least-squares model of the weighted sum of these two functions was compared with the signal in each voxel to obtain a goodness of fit statistic. The distribution of this statistic under the null hypothesis was calculated by wavelet-based resampling of the time series and refitting the models to the resampled data. Active versus control conditions were analyzed in whole brain generic group activation maps, and these were constructed by mapping the observed and randomized test statistics into standard space, and then by calculating and testing median activation maps. Use of medians prevented the interfering effects of outliers. Between-group differences were generated using a local 3D cluster analysis of activation, rather than a whole-brain 3D cluster analysis: this prevented large brain regions, such as the visual cortex from biasing the randomized null distribution and thus enabled the detection of smaller regional neural activations. Cluster-level inference with data randomization between groups was used to determine the sampling distribution of group differences under the null hypothesis. Voxel- and cluster-wise corrections (using a more stringent False Discovery Rate [FDR] threshold correction at p = 0.01 than the default p = 0.05) were applied (because of small group numbers) to ensure that data was significant at the rate of one or less false positive 3D cluster per brain.

Self-reported data for within and between-subject differences were calculated using repeated measures analysis of variance (ANOVAs), and post-hoc t-tests to confirm the direction of the differences. Examination of associations between continuous variables was calculated using the Spearman's rank non-parametric correlation coefficient (Spearman's Rho). Associations were deemed significant if correlations met the p-value threshold after Bonferroni correction.

## Results

### Sample Characteristics

Data from 8 women with BN, 18 women with AN (including 11 women with RAN and 7 women with BPAN) and 24 healthy control women were analysed. Due to time and financial constraints of the study we were unable to increase the subgroup numbers to 12 to meet the minimum p = 0.05 threshold for eighty percent power per voxel [18]. However, we emphasise that this data is prelimary and meant to generate robust hypotheses for future studies. To this aim, we applied stringent voxel and clusterwise False Discovery Rate (FDR) correction, to ensure that, despite the small sample sizes only highly significant data are reported, and give effect size data in the table. We provide both behavioural and neural evidence with p values and effect size data where possible, that the subgroups of AN are similar, and that the BPAN subgroup are more similar (in terms of effect sizes and pattern of neural activation) to the RAN group than to the BN group. Data from the AN v HC comparison analyses are presented elsewhere in unpublished work (Brooks et al., in preparation). **See Supporting Information Table S1.** Women with BN had a significantly higher (p<0.01) BMI than those with AN but were not significantly different from the HC. The mean age was 25 (7.1), in the BN group 26 (6.8), in the AN group and 26 (9.5) in HC. Women with BN had significantly more (p = 0.011) years of formal education than those with AN. Women with BN scored significantly higher (P<0.002) on the EDE-Q restrained eating scale than those with AN or HC. Additionally, women with BPAN had significantly higher restraint scores on the EDE-Q (p<0.01) than in comparison to women with BN. Women with AN and BN had a comparable mood score before the scan, but those with BN had a significantly lower (p = 0.002) mood score than HC. Furthermore, women with BPAN had a significantly higher mood than all other groups (p<0.001). The significantly higher (p<0.001) frequency of binges and vomiting per month in women with BN and BPAN compared to AN is consistent with their diagnosis. Duration of illness was not significantly different between women with AN and women with BN, and neither was it significant between the subtypes of AN.

### Subjective anxiety ratings during the scan

Women with AN and BN had comparable anxiety and depression scores, but those with BN had significantly higher (p<0.001) scores than HC. Women with AN and BN were similarly anxious during the scan when thinking about eating the food: women with BN were significantly more (p<0.001) anxious than the HC. Those women with BPAN had significantly higher anxiety ratings (p<0.001) in response to non-food images than all other groups

### Correlations between demographic variables

In the BN group, duration of illness positively correlated with BMI (Rho  =  0.829, p = 0.04) and with levels of self-reported hunger before the scan (Rho  =  0.478, p = 0.04).

### fMRI data: Within-group maps

#### Food versus non-food images

These data are shown in **Table 1** **.** In the HC group (n = 24), food images significantly increased activation in the the right insular cortex/ the right Superior Temporal Gyrus (STG) (x = 54, y = −37, z = 7, [BA 22]), the right Middle Temporal Gyrus (MTG) (x = 51, y = −37, z = 7), left side of the cerebellum (x = −4, y = −63, z = −23) and the left caudate body (x = −4, y = 0, z = 20). In the AN group (n = 18), food images significantly increased activation in the left visual cortex/cerebellum (x = −18, y = −74, z = −23, [BA 18]), the right DLPFC (x = 47, y = 7, z = 30, [BA 9]) and the right precuneus (x = 40, y = −63, z = 33, [BA 39]). In the RAN group (n = 11) food images significantly increased activation in the cerebellum (x = −25, y = −67, z = −23), left visual cortex (x = −14, y = −85, z = −13 [BA 18]), right dorsolateral prefrontal cortex (x = 40, y = 4, z = 26 [BA 9]) and parietal lobe (x = 0, y = 41, z = 46 [BA 7]). In the BPAN group (n = 7) food images signifcantly increased activation in the bilateral cerebellum (x = −4, y = −56, z = −36, x = 25, y = 63, z = 20) and right supplementary motor area (x = 22, y = −4, z = 53 [BA 6]). In the BN group (n = 8), food images significantly increased activation in the right visual cortex (x = 11, y = −81, z = −4, [BA 18], the left DLPFC (x = −33, y = 30, z = 28, [BA 9]), right insula cortex (x = −46, y = 10, z = −4, [BA 13]) and left precentral gyrus (x = −54, y = −15, z = 28 [BA3]).

**Table 1:** Within-group brain activation to food versus non-food images in, women with AN and the subtypes, BN and HC.

Brain Regions	BA	Laterality	x	y	z	Cluster size (voxels)	Cluster-p
*Food > Non-Food*							
**AN (n = 18)**							
Cerebellum	---	L	−18	−74	−23	443	0.0002
DLPFC	9	R	47	7	30	74	0.001
Precuneus	39	R	40	−63	33	119	0.002
**RAN (n = 11)**							
Cerebellum	--	L	−25	−67	−23	41	0.001
Visual Cortex	18	L	−14	−85	−13	50	0.001
DLPFC	9	R	40	4	26	66	0.002
Precuneus	7	---	0	41	46	179	0.0006
**BPAN (n = 7)**							
Cerebellum	---	L	−4	−56	−36	111	0.0008
Cerebellum	---	R	25	−63	−20	389	0.0002
SMA	6	R	22	−4	53	124	0.0009
**BN (n = 8)**							
Visual Cortex	18	R	11	−81	−4	1416	0.01
DLPFC	9	L	−33	−30	28	154	0.01
Insular Cortex	13	R	−46	10	−4	168	0.01
Precentral Gyrus	3	L	−54	−15	28	2774	0.01
**HC (n = 24)**							
Cerebellum	---	L	−4	−63	−20	497	0.0002
STG	22	R	51	−11	−7	300	0.0002
MTG	22	R	51	−37	7	78	0.002
Caudate	---	L	−4	0	20	236	0.0003

*Talairach Coordinates  =  x, Saggital plane, y, Coronal plane, z, Axial plane; Cluster size in voxels, each voxel equals 3.75mm×3.75 mm×3 mm; Cluster p = cluster probability corrected at the level of one false positive or less. ABBREVIATIONS: BA = Brodmann's Area; Laterality = L, Left, R, Right; p = probability; AN = Anorexia Nervosa, RAN = Restricting Anorexia Nervosa, BPAN = Binge Purge Anorexia Nervosa, BN = Bulimia Nervosa, HC = Healthy Controls; DLPFC = Dorsolateral Prefrontal Cortex, SMA = Supplementary Motor Area; STG = Superior Temporal Gyrus, MTG = Middle Temporal Gyrus.*

### fMRI data: Between-group comparisons

#### Activation to food images compared to non-food images

**Figure 1:**
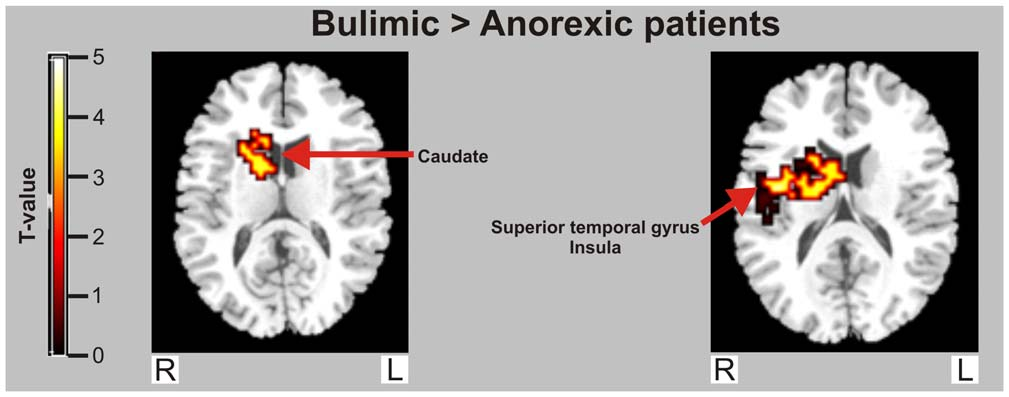
Between-group map activation showing food > non-food activation in women with bulimia nervosa versus anorexia nervosa. T-value bar illustrates t-value scores represented by cluster on the brain map.

Data are shown in Fig 1. and Table 2. In comparison to the HC group, the BN group did not show significantly greater activations to food images, but showed relative deactivation in the bilateral superior temporal gyrus/insular cortex (x = 54, y = −26, z = −7[BA 38/13] / x = −51, y = −15, z = 0 [BA 22/13]), and left visual cortex (x = 44, y = −67, z = 26 [BA31]).

**Table 2:** Between group contrast activation to food images in women with AN, BN and HC.

Brain Regions	BA	Laterality	x	y	z	Cluster size (voxels)	Cluster-p
*Food > Non-Food*							
**AN>BN**							
Parietal lobe	2	R	54	−26	−35	43	0.003
PCC	31	L	−11	−52	46	13	0.006
**RAN>BN**							
Precentral Gyrus	6	R	43	−4	43	20	0.008
**BPAN>BN**							
ITG	37	L	−36	−56	−17	33	0.008
**BN>AN**							
Caudate	---	R	40	−4	13	48	0.004
STG/Insula	22	R	14	7	21	68	0.005
SMA	6	L	−43	4	43	57	0.003
**BN>RAN**							
ITG	21	L	−58	−4	−10	7	0.008
Fusiform Gyrus	18	L	−4	−67	−10	35	0.005
PCC	23	---	0	−41	13	43	0.005
ITG	21	R	47	−37	36	62	0.001
IPL	7	L	−4	−52	46	41	0.001
**BN>BPAN**							
Cerebellum	---	L	−18	−63	−40	29	0.004
PHG	28	L	−22	4	−26	12	0.004
PCC	31	L	−4	−67	26	23	0.008
SMA	6	R	25	7	53	32	0.008
**BN>HC**							
**HC>BN**							
STG/Insula	38/13	L	54	−26	−7	18	0.001
STG/Insula	22/13	R	−51	−15	0	17	0.001
Visual Cortex	31	L	44	−67	26	12	0.002

*Talairach Coordinates  =  x, Saggital plane, y, Coronal plane, z, Axial plane; Cluster size in voxels, each voxel equals 3.75 mm×3.75 mm×3 mm; Cluster p = cluster probability corrected at the level of one false positive or less. ABBREVIATIONS: BA = Brodmann's Area; Laterality = L, Left, R, Right; p = probability; AN = Anorexia Nervosa, RAN = Restricting Anorexia Nervosa, BPAN = Binge Purge Anorexia Nervosa, BN = Bulimia Nervosa, HC = Healthy Controls; PCC = Posterior Cingulate Cortex; ITG = Inferior Temporal Gyrus; STG = Superior Temporal Gyrus; SMA = Supplementary Motor Area; IPL = Inferior Parietal Lobe; PHG = Parahippocampal Gyrus;*

Compared to the AN group, the BN group had relatively reduced activation to food images in the right parietal lobe (x = 54, y = −26, z = 35 [BA 2], and left dorsal posterior cingulate cortex (x = −11, y = −52, z = 46 [BA 31]), and increased activation in the right caudate (x = 40, y = −4, z = 13), right superior temporal gyrus (x = 14, y = 7, z = 21 [BA 22]) and left supplementary motor area (x = −43, y = 4, z = 43 [BA 6]).

Compared to the RAN group, the BN group showed relatively increased activation in the bilateral inferior temporal lobe (x = −58, −4, −10; x = 47, y = −37, z = 36 [BA 21]), left visual cortex (x = −4, −67, −10 [BA 18], posterior cingulate (x = 0, y = −41, z = 13 [BA 23]), and the left inferior parietal lobe (x = −4, y = −52, z = 46 [BA 7]). The BN group compared to the RAN group showed relative deactivation to food images in the right precentral gyrus (x = 43, y = −4, z = 43 [BA 6]).

Compared to the BPAN group, the BN group had relatively greater activation in the left cerebellum (x = −18, y = −63, z = −40), left parahippocampal gyrus (x = −22, y = 4, z = −26 [BA 28]), left posterior cingulate cortex (x = −4, y = −67, z = 26 [BA 31]), right supplementary motor area (x = 25, y = 7, z = 53 [BA 6]). The BN group had deactivation relative to the BPAN group in the left inferior temporal gyrus (x = −36, y = −56, z = −17 [BA 37]).

### fMRI data: effects of SSRIs

In the AN group, 10/18 women were on SSRI medication. As this might alter neural activation [19] independent t-tests were run to check whether differences occurred in neural activation between those were/ were not taking SSRIs. This was done in areas where neural activation in response to food images were significantly different from HC. There were no significant differences between those who were taking SSRIs and those who were not.

### fMRI data: correlations between neural activation and participant demographics

We found no significant correlations between any significant clusters of neural activation and participant demographics.

## Discussion

Using a novel fMRI paradigm, whereby participants thought about eating food shown in images rather than passively viewing them, we have compared neural responses in women with BN and AN. These exploratory data provide the first evidence that patterns of food consumption (e.g. binges) combined with degree of restraint over eating behaviour may alter how the brain responds when thinking about eating food. However, caution must be exercised; despite stringent threshold corrections, the small sample sizes make these intriguing results somewhat tentative at this stage, and in need of further clarification with larger cohorts. Activation of the dorsolateral prefrontal cortex, visual cortex and cerebellum in response to food versus non-food images was found in women with AN and BN. Additionally, women with BN show greater somatosensory and motor responses in the right insular cortex and post-central gyrus. Women with BN showed increased activation in somatosensory, motor and appetitive regions in comparison to AN and HC. Furthermore, women with BPAN had reduced activation in comparison to women with BN in regions associated with appetitive and motor responses. Thus, although women with BPAN partake in binge eating and purging behaviour in a similar way to women with BN, their heightened control over eating (as shown by an emaciated body at less than 85 percent of normal body weight) may be achieved by a reduced drive to eat over the long term. Thus, these results suggest a clear BN-specific map of brain activation in response to thinking about eating food shown in images, which is primarily characterized by signs of increased appetitive, somatosensory and motor responses, in parallel with activation of prefrontal cortex cognitive inhibition. However, these intriguing suggestions need to be tested further.

Activation of regions associated with motor responses in women with BN, such as the caudate, supplementary motor area and the precentral gyrus suggests an increased appetitive response to food images. This pattern of activation accords with other fMRI studies of people who binge-eat [4], [7], [9], [20]. Increased motor responses to food images may reflect an anticipated desire for food consumption, as these regions are also activated in healthy people who have fasted and report a craving for food [21], [22], [23], [24]. Furthermore, activation of the caudate and motor regions (precentral gyrus, SMA) are implicated in addictive behaviour [25], [26] and thus might suggest that there are some similarities in neural systems between addiction and binge eating. Our data suggest that women with BN have an elevated neural appetitive response to food images, combined with activation of motor areas, when thinking about eating food.

We also observed an increased right dorsolateral prefrontal cortex (DLPFC) response to the food versus non-food images, but this was comparable in both eating disordered groups (there was no significant differential DLPFC activation when women with AN and BN were compared). The DLPFC forms part of the cognitive control network, and is associated with restriction of appetitive responses [27], [28], [29]. Activation of a “top-down” cognitive control network, which interacts with “bottom-up” appetitive responses (e.g. the striatum) might be expected when women, who have a pathological desire to be thin and to control their food intake, think about eating food. This is likely to be sporadic in women with BN, who attempt to control their food intake but who are prone to yield to binge eating. In line with this notion, it is plausible that, in women with BPAN, intermittent activation of appetitive systems impinge on attempts at top-down control, but to a lesser extent than those with BN. It is currently unclear however, whether it would be greater top-down control or weaker bottom-up activation that prevents a person with BPAN from gaining weight and/or developing BN symptoms.

The balance between these behaviours is likely dependent on the level of bottom-up impingement on cognitive control, as has been shown in two studies with conflicting results: one shows greater, while the other reduced DLPFC activation (Lock et al., 2011; Marsh et al., 2009, respectively). Both studies examined response inhibition in females with BN, one study using the ‘Go/No-Go’ task [30], the other using the ‘Simon Incompatibility’ [31]task, which may activate different levels of arousal (particularly during incorrect responses), and which would account for differential disruption to PFC networks. Additionally, women who have recovered from BN continue to show an aberrant pattern of activation in the striatum in response to reward[9] and also a reduced pattern of activation in PFC regions to the taste of glucose [8]. It could be that as a woman with BN recovers, the arousal system in the brain, albeit aberrant, is tamed and causes less impingement on PFC cognitive networks. Furthermore, there could be different aetiologies for women with AN and BN, in that the former is driven by excessive activation of PFC cognitive inhibition systems, whereas the latter is driven by excessive arousal in reward regions. This could explain some of the differential neural activation observed between women with AN and BN in this study.

The insular cortex functions as an intermediary between the PFC and striatum activation, with dense functional connectivity between these regions, creating an emergent sentience of bodily state [32]. Furthermore, dysregulation of the insula seems to be prominent in the pathophysiology underlying neural activation in people with eating disorders [33], [34]. We have observed increased insula activation during the food versus non-food contrast in the BN and the HC groups, but not in the AN group. However, in comparison to HC, those with BN have reduced bilateral insula activation in response to food stimuli. These data suggest that there is an intact, perhaps heightened awareness of appetitive responses in women with BN, which is comparatively reduced in women with AN.

Both BN and AN groups demonstrated an increased visual cortex response during the food versus non-food contrast. However, women with BN had a reduced visual cortex response in comparison to HC. Visual cortex activation usually reflects attention towards the stimuli. Studies of attentional bias using the Stroop task show that women with BN have a greater attentional bias to food stimuli than HC [35]. However, Stroop studies do not demonstrate whether the attentional bias reflects hypervigilance towards, or avoidance of stimuli. The reduced visual cortex activation to food images (in comparison to HC) observed here suggests that the BN group may have been using strategies to cognitively avoid processing the food stimuli. It is unlikely that BN subjects were closing their eyes because there was differential visual cortex activation in the food versus non-food contrast. However, cognitive avoidance strategies are likely to be a method whereby women with BN can circumvent their appetitive drive in order to achieve self-restraint, which might be reflected in reduced visual cortex activation.

Additionally, the women with BN had significantly higher restraint scores (EDE-Q) than those with AN, and the women with BPAN had the highest restraint score, which is in line with observations that people who binge are often relapsing from attempts at restraint [36] But this may also reflect and support a recent finding that the EDE-Q is less effective at identifying bulimic-type eating behaviours[37]. For example, a high restraint score on the EDE-Q may indicate subjective feelings of restraint, when in reality restraint of food intake is low. Despite the EDE-Q being an excellent tool, perhaps there is a now a need to re-evaluate the validity of some of the constructs tested, particularly in relation to binge eating behaviour.

It would be of benefit in future fMRI studies to compare the neural responses to high versus low calorie food images, as it has not been done in bulimic women. In healthy women it has been shown that images of high calorie foods activate brain regions associated with somatosensory responses and reward more so than compared to low calorie food images [38]. This suggests that the images are cognitively evaluated for food palatability, associated with top-down PFC activation, which modulates responses in reward regions in the brain (e.g. in the striatum). Women with BN show dysregulation in a fronto-striatal neural circuit [5] and so it would add to existing neural models of bulimia to compare high and low calorie food images in this group.

Additionally, future studies should ensure a totally homogeneous sample of AN women and a much larger sample size, unlike in this preliminary study, where we include 11 restricters and 7 binge-purgers in our total AN group. It could be argued that the binge-purge AN subtype bear similarities with the BN group, by the very nature that both groups partake in binge eating behaviour. However, a counter-argument is that, based on the observation that they were severely underweight, all the women with AN in this study (regardless of subtype) were more successful at restricting their food intake over the long-term than the women with BN. This was also reflected in reduced activation of appetitive and motor brain regions to food images in the women with BPAN compared to BN, and that differences on behavioural measures were comparable between BN v BPAN and between BN v RAN.However, one solution would have been to remove the binge-purgers from our total AN group. However, by including the broad range of AN pathologies we can pinpoint in this cohort the one factor they have in common: restrained eating behaviour that results in self-starvation and a dramatic loss of weight. Thus, we have compared an inhomogeneous group of AN women (who all restrict their food intake) with a group of BN women, to localise differential neural activation associated with long-term restriction of food intake.

There are some limitations to this study: the sample size in the BN group is small (n = 8), and so caution must be exercised when generalising to the BN phenotype. However, in an attempt to overcome the small group numbers, we took the relatively uncommon step of using stringent False Discovery Rate (FDR) correction at both the voxel and cluster level to ensure a rate of a maximum of one false positive. In addition, we provide effect sizes for all demographic data. Furthermore, we did not examine differential neural responses to high versus low calorie food images: by doing this we could examine activation to stimuli of different motivation value. Moreover, unlike in this study, future studies should include a homogeneous and larger sample of women with AN, and also men It is entirely plausible that the small percentage of men who suffer from AN and exhibit the same symptoms also show similar patterns of neural activation to food images. Finally, we used the EDE-Q rather than the EDE to gauge eating disorder pathology, which has been shown to be a good measure overall, but weaker for bingeing behaviour [37]. However, we chose to use the EDE-Q to reduce the length of the experiment for each participant, as the EDE is a longer interview version that must be completed on a one-to-one with the researcher. A longer experimental duration would have had confounding effects on neural activation (e.g. fatigue), and the EDE-Q was sufficient for our purposes to support eating disorder diagnoses derived initially from the SCID.

The data from this and other studies allow the following tentative conclusions that are in need of further testing. People with BN have increased appetitive and motor responses to food stimuli in parallel with varying (and perhaps sporadic) levels of cognitive inhibition arising from the DLPFC. An imbalanced convergence on the insular cortex between these cortical and subcortical processes alters interoceptive awareness and contributes to binge eating, purging and disrupted self-regulation. Future fMRI studies of BN should seek to confirm these findings and to extend knowledge of the interactions between the DLPFC, insular cortex and caudate, perhaps by using dynamic causal modelling (DCM).

## Supporting Information

Table S1 Demographic characteristics and self-report measures: demonstrating the mean values, standard deviations, and differences in scores between anorexic and bulimic women.
(DOCX)
