# Supplementary material for: Differential Neural Responses to Food Images in Women with Bulimia versus Anorexia Nervosa
Source: PLoS One. 2011 Jul 20;6(7):e22259. doi: 10.1371/journal.pone.0022259 (PMC3140495; doi:10.1371/journal.pone.0022259)
Supplement: Table S1 — Demographic characteristics and self-report measures: demonstrating the mean values, standard deviations, and differences in scores between anorexic and bulimic women. (DOCX) [file pone.0022259.s001.docx]

Table S1. Demographic characteristics and self-report measures: demonstrating the mean values, standard deviations, and differences in scores between anorexic and bulimic women

| a) |  |  | Means, SDs |  |  |  |  |  | Contrast | Effect Sizes: | Cohen’s *d* |  |  |
| --- | --- | --- | --- | --- | --- | --- | --- | --- | --- | --- | --- | --- | --- |
|  | AN(n=18) | RAN(n=7) | BPAN(n=7) | BN(n=8) | HC(n=24) | ANvBN | BNvHC | ANvHC | RANvHC | BPANvHC | RANvBN | BPANvBN | RANvBPAN |
| **Age,** years | 26(6.8) | 26(7.2) | 25(6.6) | 25(7.1) | 26(9.5) | 0.16 | 0 | 0 | 0.02 | 0.07 | 0 | 0.16 | 0.06 |
| **BMI,** kg/m² | 15.7(1.2) | 15.2(1.2) | 16.4(0.6) | 21.6(3.4) | 21.7(2.4) | 3.0 | 0.04 | 3.1 | 2.93 | 2.66 | 1.22 | 0.9 | 1.22 |
| **Education,** years | 8(3) | 8(4) | 8(3) | 12.5(6.3) | 12(6.5) | 1.1 | 0.08 | 0.76 | 0.7 | 0.76 | 0.87 | 0.98 | 0.14 |
| **Duration of ED,** years | 7.2(4.0) | 9(7.4) | 9(6.1) | 12(7.8) | - | 0.9 | - | - | - | - | 0.45 | 0.5 | 0.01 |
| **SSRI medication** (No., %) | 10(55.5) | 4(36) | 6(86) | - | - | - | - | - | - | - | - | - | 0.04 |
| **EDE-Q (0-6)** |  |  |  |  |  |  |  |  |  |  |  |  |  |
| - restrained eating | 2.6(1.7) | 2.1(1.6) | 5.5(1.8) | 4.4(0.3) | 0.8(1.0) | 1.3 | 4.2 | 1.81 | 1.1 | 1.2 | 1.76 | 3.31 | 0.92 |
| **EDE-Q No. of binges in a month:(Q8, 0-6)** | 0.9(1.78) | - | 2(3) | 4.4(1.6) | - | 2.1 | - | - | - | - | 0.16 | 2.54 | - |
| **EDE-Q No. of vomits in a month: (Q22)** | 2.8 (7.28) | - | 7(10) | 18.6(10.6) | - | 2.0 | - | - | - | - | - | 0.07 | - |
| **HADS- Anxiety (0-21)** | 13.6(3.6) | 12(9) | 17(3) | 11.2(3.8) | 4.4(2.7) | 0.7 | 2.1 | 4.19 | 2.7 | 2.8 | 0.88 | 0.88 | 1.14 |
| **HADS- Depression (0-21)** | 13.6(3.7) | 8(5) | 12(9) | 7.2(4.3) | 1.6(1.7) | 1.7 | 2.0 | 4.5 | 2.9 | 2.7 | 0.22 | 0.69 | 0.88 |
| **Psychiatric co-morbidity (No., %)** | 14(77.7) | 8(78) | 6(71) | 1(11.1) | - | 0.7 | - | - | - | - | 0.12 | 0.11 | 0.04 |
| - Depressive disorders (No., %) | 3(16.6) | 2(18) | 1(14) | - | - | - | - | - | - | - | - | - | 0.06 |
| - Anxiety disorders (No., %) | 6(33.3) | 8(27) | 2(29) | 1(11.1) | - | 1.7 | - | - | - | - | 0.34 | 0.05 | 0.04 |
| - Depression & Anxiety (No., %) | 5(27.7) | 8(27) | 2(29) | - | - | - | - | - | - | - | - | - | 0.04 |
| b) |  |  | Means,SDs |  |  |  |  |  | Contrast | Effect Sizes: | Cohen’s *d* |  |  |
|  | AN(n=18) | RAN(n=7) | BPAN(n=7) | BN(n=8) | HC(n=24) | ANvBN | BNvHC | ANvHC | RANvHC | BPANvHC | RANvBN | BPANvBN | RANvBPAN |
| **Mood (0-10)** | 4.2(2.2) | 4.47(2.3) | 8.91(2.2) | 3.6(2.2) | 6.4(1.7) | 0.28 | 1.58 | 0.28 | 1.05 | 1.43 | 0.41 | 2.59 | 2.08 |
| **Food anxiety (0-10)** | 6.9(1.8) | 6.98(2.1) | 7.66(2.0) | 6.5(1.5) | 2.4(2.0) | 0.24 | 2.23 | 0.24 | 2.32 | 2.72 | 0.27 | 0.71 | 0.35 |
| **Non-food anxiety (0-10)** | 1.8(0.9) | 1.44(0.78) | 2.68(0.48) | 1.0(1.1) | 1.8(1.7) | 0.86 | 0.75 | 0.86 | 0.25 | 0.59 | 0.5 | 2.07 | 1.93 |
| **Aversive anxiety (0-10)** | 8.2(1.5) | 7.91(1.61) | 8.67(1.2) | 8.0(1.1) | 7.3(2.3) | 0.15 | 0.35 | 0.15 | 0.3 | 0.67 | 0.07 | 0.63 | 0.55 |
| **Neutral anxiety (0-10)** | 2.1(2.4) | 1.70(1.79) | 2.97(8.9) | 1.6(1.3) | 1.5(1.3) | 0.24 | 0.08 | 0.24 | 0.14 | 0.36 | 0.07 | 0.24 | 0.24 |

Table S1. a) gives information about main demographic data; table b) gives information about self-report ratings of mood before the scan, and self-report ratings of anxiety during the scan. Values expressed as mean, standard deviations(S.D.); ABBREVIATIONS: AN=Anorexia Nervosa; RAN= Restricting Anorexia Nervosa; BPAN=Binge Purge Anorexia Nervosa; BN= Bulimia Nervosa; HC= Healthy Control; BMI=Body Mass Index; ED=Eating Disorder; anx=anxiety measured during the scan as a self-report verbal response where 0=least anxious and 10=most anxious; SSRI=Selective Serotonin Reuptake Inhibitor; EDE-Q=Eating Disorder Examination Questionnaire, 0-6 scale for subscale scores where 0 is least severe, 6 is most severe, EDE-Q (Q8) number of binges in one month, scale 0= no days, 1=1-5 days, 2=6-12 days, 3=13-15 days, 4=16-22 days, 5=23-27 days, 6=everyday, EDE-Q (Q22) number of vomits in month=absolute number of vomits in one month, HADS=Hospital Anxiety and Depression Scale, 0-21 scale where 0 is least severe and 21 is most severe for trait anxiety and depression as subscales; effect sizes for the contrasts are calculated using Cohen's *d*.
